# 

**Published:** 1904-10-08

**Authors:** 


### NOTICE TO CORRESPONDENTS.

All MSS., Letters, Books for Review, and other matters intended for the Editor should be addressed The Editor, "The Hospital," The Hospital Buildings, 28 & 29 Southampton Street, Strand, W.C.

The Editor cannot undertake to return rejected MS., even when accompanied by stamped directed envelope.

**Notice to Advertisers and Subscribers.**

All Advertisements, Orders for Copies of The Hospital, and Business Communications should be addressed to **THE MANAGER (Not to the Editor)**, The Hospital, 28 & 29 Southampton Street, Strand, London, W.C.

The Cost of Subscription, post free, is as follows:—Per week, 3½d.; per quarter, 3s. 3d.; per half-year, 6s. 6d.; per annum, 13s. Foreign and Colonial:—Per annum, 19s. 6d., payable, in all cases, in advance.

**NOTICE.—Vol. XXXV. of "The Hospital" (October 1903 to March 1904), in handsome cloth binding, is now on sale at the office of this Journal, price 10s. 6d. Cases for binding the Numbers contained in this Volume 2s. Index to Vol. XXXV., 3½d., post free.**

**The Monthly part for October is now ready. Price 1s., by post, 1s. 3d.**

### MEDICAL APPOINTMENTS.

J. S. Hudson, M.R.C.S., L.R.C.P.Lond., District Medical Officer of the Watford Union. **Wm. Crosby Johnson**, M.B., Ch.B.-Vict., Honorary Physician to the Pendleton Branch Dispensary of the Salford Royal Hospital. **C. W. Low**, M.B.Durh., Medical Officer of Health, Thedwastre Rural District. **Frank Mathews**, L.R.C.P.Lond., M.R.C.S.Eng., Medical Officer for the Nantwich District of the Nantwich Union. **Harry Overy**, M.B., Ch.B Edin., F.R.C.S.Eng., Casualty Officer and Pathologist to the Metropolitan Hospital, N.E. **W. L. Pethybridge**, M.D., B.Sc.Lond., Honorary Physician to the Plymouth Public Dispensary, Honorary Pathologist to the South Devon Hospital, and Ophthalmologist to the Plymouth Education Authority. **T. R. Rodger**, M.B., Ch.B.-Glasg., Certifying Factory Surgeon for the Sanquhar District, Dumfries. **John Round**, L.R.C.P., L.R.C.S., Physician's Assistant to the Plymouth Public Dispensary. **H. Wilcox**, M.B.Aberd., M.R.C.S.Eng., Medical Officer of Health, Fleet Urban District. **J. C. Williams**, M.R.C.S., L.R.C.P.Lond., Medical Officer of Health, Barmouth Urban District.

Bartholomew's Hospital.—The following appointments have been made:—House Physicians: **C. M. H. Howell, A. R. Neligan, J. E. Payne, C. H. D. Adams, H. V. Wenham** (*Senior*); **E. H. White, B. Hudson, C. A. Anderson, F. A. Hepworth, C. F. Hadfield** (*Junior*). House Surgeons: **H. D. Ledward, H. W. Wilson, R. B. Etherington-Smith, H. N. Burroughes, T. B. A. Haggard** (*Senior*); **G. H. Colt, H. J. D. Birkett, C. T. H. Plowright, L. Cripps, J. Burfield** (*Junior*), Resident Midwifery Assistant: **H. A. Gould.** Ophthalmic House Surgeon: **L. Noon.** Assistant Chloroformists: **W. F. Cross** and **H. E. G. Boyle.** Extern Midwifery Assistants: **N. Macfadyen** and **A. H. Hogarth.** Chief Assistants: (Department for Diseases of Women) **H. Williamson**, M.B.Cantab., M.R.C.P. (Department for Diseases of the Eye) **E. W. Brewerton**, F.R.C.S. (Department for Diseases of the Ear) **C. E. West**, M.B.Oxon., F.R.C.S.; **S. R. Scott**, M.B., B.S.Lond., F.R.C.S. (Department for Diseases of the Throat and Nose) **W. Jobson Horne**, M.D.Cantab.; **F. A. Rose**, M.B.-Cantab., F.R.C.S. (Department of Orthopædics) **G. E. Gask**, F.R.C.S. (Department for Diseases of the Skin) **T. J. Horder**, M.D., B.Sc.Lond., M.R.C.P.; **H. Thursfield**, M.D.Oxon, M.R.C.P. (Department of Electro-Therapeutics) **H. Walsham**, M.D.-Cantab., F.R.C.P.; **J. M. Flavelle**, M.R.C.S. (Dental Department) **F. Coleman**, M.R.C.S., L.D.S.; **B. Stevenson**, L.D.S.-Glasg.

**"The Hospital" Institutional Library.**

**"Hospitals and Asylums of the World." 4 Vols., with a Portfolio of Plans, complete £12 12s.**

**"Burdett's Hospitals and Charities, 1904." 5s. net.**

**"Burdett's Official Nursing Directory." 3s. net.**

**"Cottage Hospitals, General, Fever, and Convalescent." 10s. 6d.**

**"Uniform System of Accounts for Hospitals and Public Institutions." New Edition. 4s. net.**

**"Hospital Expenditure: The Commissariat." 2s. 6d.**

**"A Legal Handbook for the Use of Hospital Authorities." 1s. 6d.**

**"The Cottage Hospital Case Book or Register of Patients." 10s. and 12s. 6d.**

**All these are published by the Scientific Press, Ltd., and may be obtained through any bookseller, or direct from the publishers, 28 and 29 Southampton Street, Strand, London, W.C.**

### Medical Appointments.

An EXAMINATION OF CANDIDATES for entry into the Medical Department of the Royal Navy, will be held on 14th November next and following days at Examination Hall, Thames Embankment.

The forms to be filled up by Candidates will be supplied on application to the Medical Director-General.

Admiralty,

18 Victoria Street, S.W.

30th September, 1904.

(53)

### NURSING LITERATURE.

### Matrons.

**HOSPITAL EXPENDITURE.** A Treatise on Domestic Expenditure and the Commissariat. Price **2/6** post free.

**SYLLABUS OF LECTURES TO NURSES.** A Valuable Aid in Preparing Lectures. Price **1/-** post free.

**LINEN REGISTER.** Ruled for Stock Condemned; Remaining and Issued Linen; also Total in Ward, and Remarks; also Lists of Hospital Linen. Price **7/6** post free.

**Also Case-Books, Account Books, and Charts of all Descriptions. Particulars and Prices on application.**

### Sisters.

**HOSPITAL SISTERS AND THEIR DUTIES.** By the Matron of the London Hospital. Price **2/6** post free.

### Hospital Nurses.

**A HANDBOOK FOR NURSES.** By Dr. J. K. Watson, M.D. Price **5/-** net; **5/4** post free.

**THE NURSES' PRONOUNCING DICTIONARY.** By Honnor Morten. Fifth Edition. Revised and Edited by Mary I. Burdett. **2/-** post free.

### Private Nurses.

**THE ART OF FEEDING THE INVALID.** Price **1/6** post free.

**MODERN NURSING IN PRIVATE PRACTICE.** By Sir William Bennett. Price **6d.** net; post free **7d.**

**PREPARATION FOR OPERATION IN PRIVATE HOUSES.** **6d.** post free.

### District Nurses.

**PRACTICAL HINTS ON DISTRICT NURSING.** By Amy Hughes. Price **1/-** post free.

**THE "SIMPLEX" DISTRICT NURSES' CASE-BOOK.** Price **6d.** net; post free **7d.**

### Midwifery Nurses.

**A COMPLETE HANDBOOK OF MIDWIFERY.** By J. K. Watson. The best and latest book on the subject. **6/-** net; **6/4** post free.

**MIDWIVES' POCKET BOOK.** By Honnor Morten. **1/-** post free.

*Send for New Catalogue of Nursing Manuals, Case-Books, &c., post free on application.*

**THE SCIENTIFIC PRESS, LTD.,** 28 & 29 Southampton St., Strand, London, W.C.

